# Primary Roles of Branched Chain Amino Acids (BCAAs) and Their Metabolism in Physiology and Metabolic Disorders

**DOI:** 10.3390/molecules30010056

**Published:** 2024-12-27

**Authors:** Tomoki Bo, Junichi Fujii

**Affiliations:** 1 Laboratory Animal Center, Institute for Promotion of Medical Science Research, Faculty of Medicine, Yamagata University, Yamagata 990-9585, Japan; 2 Department of Biochemistry and Molecular Biology, Graduate School of Medical Science, Yamagata University, Yamagata 990-9585, Japan

**Keywords:** branched chain aminotransferase, branched chain α-keto acid dehydrogenase, ketogenesis, gluconeogenesis, diabetes, cancer

## Abstract

Leucine, isoleucine, and valine are collectively known as branched chain amino acids (BCAAs) and are often discussed in the same physiological and pathological situations. The two consecutive initial reactions of BCAA catabolism are catalyzed by the common enzymes referred to as branched chain aminotransferase (BCAT) and branched chain α-keto acid dehydrogenase (BCKDH). BCAT transfers the amino group of BCAAs to 2-ketoglutarate, which results in corresponding branched chain 2-keto acids (BCKAs) and glutamate. BCKDH performs an oxidative decarboxylation of BCKAs, which produces their coenzyme A-conjugates and NADH. BCAT2 in skeletal muscle dominantly catalyzes the transamination of BCAAs. Low BCAT activity in the liver reduces the metabolization of BCAAs, but the abundant presence of BCKDH promotes the metabolism of muscle-derived BCKAs, which leads to the production of glucose and ketone bodies. While mutations in the genes responsible for BCAA catabolism are involved in rare inherited disorders, an aberrant regulation of their enzymatic activities is associated with major metabolic disorders such as diabetes, cardiovascular disease, and cancer. Therefore, an understanding of the regulatory process of metabolic enzymes, as well as the functions of the BCAAs and their metabolites, make a significant contribution to our health.

## 1. Introduction

Amino acids are the sources of biological components such as proteins, nucleic acids, and bioactive amines in anabolic reactions, and they also are the primary energy substrates in catabolic reactions during both periods of fasting and sustained exercise. Among hundreds of amino acids, twenty are considered to be the building blocks of proteins, and these are metabolized according to corresponding nutritional requirements. In humans, almost half of the proteinaceous amino acids cannot be synthesized from metabolic intermediates and must be consumed in food [1,2]. Leucine (Leu), isoleucine (Ile), and valine (Val) are essential amino acids and are collectively referred to as branched chain amino acids (BCAAs) because they have branched structures in their side chains and uniquely share two of the initial metabolic pathways. Muscular proteins are dominantly built with BCAAs, which make up 20% to 25% of all amino acids in dietary proteins and play primary roles in muscular function [1,2].

The first process of BCAA catabolism catalyzed by branched chain aminotransferase (BCAT) and the second process catalyzed by branched chain α-keto acid dehydrogenase (BCKDH) are common to all three BCAAs [3,4]. BCAAs are rarely metabolized by the liver because the expression of BCAT, which acts to remove amino groups in the first step of any amino acid catabolism, is very low. This metabolic property allows dietary BCAAs to be delivered effectively in intact forms to muscular tissues [2]. At that point, muscular cells use BCAAs along with other amino acids to synthesize proteins under regulation by the mechanistic targeting of rapamycin (mTOR) [5]. By contrast, under either fasting conditions or sustained exercise, muscular proteins are selectively degraded, which occurs mainly via the ubiquitin-proteasome system (UPS) and by macro autophagy [6,7,8,9]. The resultant amino acids and their metabolites are used partially by muscles themselves but are largely secreted to meet the energy demands of other organs through the processes of gluconeogenesis and ketogenesis in the liver. In this manner, muscles are believed to function not only as a locomotor organ but also as a reservoir for amino acids in the form of proteins. Inborn errors of genes during BCAA catabolism could result in a rare inherited condition referred to as the maple syrup urine disease (MSUD) [10,11]. Moreover, BCAA metabolism is associated with major metabolic disorders such as diabetes, cardiovascular disease, and cancer, which has resulted in BCAA metabolism being considered a potential target for pharmacological treatment [12,13,14,15]. On the other hand, BCAAs could produce beneficial effects on aging and are associated with longevity in animals, but there is no consensus on these issues [16,17,18,19,20].

In this article, we overview the unique properties of BCAA degradation and discuss the significance of BCAA metabolism in metabolic disorders with a particular focus on the regulation of metabolic enzymes and their gene expression as well as on the associated pathological conditions—the most noteworthy being cancer.

## 2. Nutritional Aspect of BCAA Metabolism Under Conditions of Fasting and Exercise

Since amino acid metabolism varies depending on the organs, cells, and the nutritional state of an individual, the roles of this metabolism must be understood from the perspective of biochemical reactions and gene expression under individual physiological situations. Among essential amino acids, BCAAs are abundantly required for constructing muscle proteins during growth in children and for athletes engaging in sustained exercise, which means that a sufficient intake of BCAAs in the diet is highly recommended. Upon fasting or sustained exercise, muscular proteins undergo proteolysis, which releases BCAAs as a dominant energy source. In contrast, high concentrations of BCAAs and their metabolism in blood are associated with obesity and diabetes [13,14,21]. Hence, understanding the metabolism of BCAAs is of primary importance not only for daily life but also for maintaining proteostasis during physical exercise and under conditions of metabolic disease [5,22].

### 2.1. Processes for BCAA Absorption in the Luminal Side of the Intestine and Their Delivery to Major Organs

Most nutrients are absorbed in the intestine, and membrane transporters are required for both taking up and secreting hydrophilic compounds, such as carbohydrates and amino acids/small peptides [23]. Gene expression that encodes transporter proteins primarily determines how cells uptake, utilize, and secrete them. Due to the presence of numerous nutritional compounds and for reasons of a historical nature, the nomenclature of amino acid transporters is complex. Some amino acid transporters were first classified and named based on pharmacological or kinetical properties before the corresponding proteins or genes were actually identified. Subsequent studies have identified proteins and their genes, which led to the adoption of unique names by researchers—often in an asystematic manner. For some transporters, the order of their naming and the elucidation of their functions is reversed; i.e., genes with unknown functions were found first, after which their functions were identified in subsequent studies. Genes for the transporters are now named using a more systematic procedure [see SLC Tables http://slc.bioparadigms.org/, accessed on 1 October, 2024]. Accordingly, one amino acid transporter could be associated with different names, which is sometimes confusing. In this article, we will mainly use the conventional name of the main protein component responsible for the amino acid transport but also occasionally mention the gene name when discussing their expressions and mutations.

**Figure 1:**
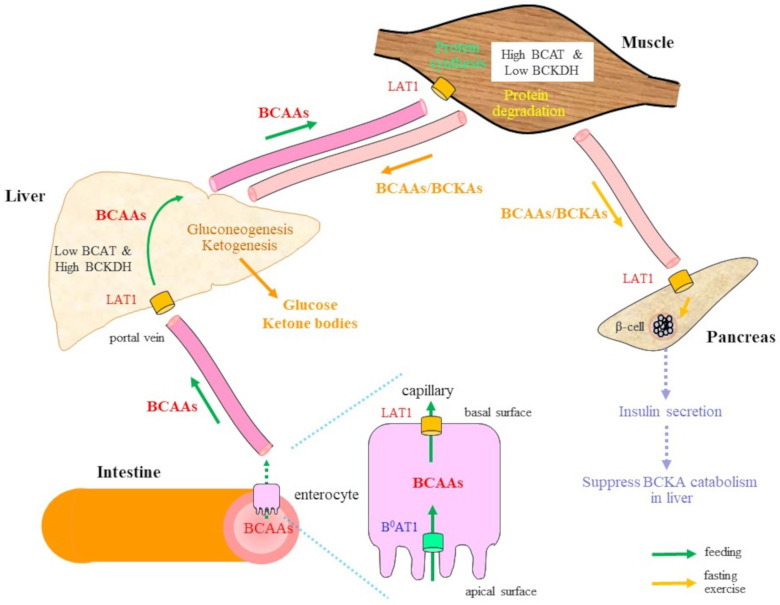
Intestinal absorption of BCAAs and their subsequent dynamics in major organs. Dietary BCAAs are absorbed by B^0^AT1 on the apical surface of intestinal epithelial cells and then are secreted into the blood by LAT1 on the basal surface. BCAAs enter the liver through the portal vein, but because BCAT activity is low, they are not metabolized in the liver. BCAAs, in turn, enter blood circulation, where they are mainly taken up by muscles and used for protein synthesis. It is believed that LAT1 is mainly responsible for the uptake of BCAAs from the blood into cells. On the contrary, during periods of either nutritional starvation or intense exercise, muscle protein is degraded, and BCAAs are preferably metabolized in muscle cells. It appears significant, however, that a portion of BCKAs is secreted into the bloodstream and taken up by the liver as well as by other organs. Some BCKAs are reportedly involved in insulin secretion from pancreatic β-cells. Since the metabolism of BCAAs is the main subject of this article, readers should refer to the text for details.

In intestinal epithelia, amino acid transporters associated with the apical membrane differ from those at the basolateral membrane (Figure 1), which is a situation also seen with respect to carbohydrate transporters [24,25]. Transporters of the apical membrane generally utilize energy to uptake amino acids by employing mechanisms such as the sodium concentration gradient and proton motive force. After secretion into the bloodstream via passive transporters, amino acids, along with other hydrophilic nutrients, are delivered to the liver via the portal vein. The size, hydrophobicity, and charge of the amino acid side chains are the main factors that determine the pairing with a transporter. Individual amino acids are generally carried by more than one transporter, with some preferences for their partners.

Genes classified into the solute carrier (SLC) superfamily account for approximately 20% of human genes encoding membrane proteins [26]. Sixty-six amino acid transporters belong to eleven SLC families. BCAAs, along with methionine (Met), tryptophan, and threonine in nutrients, are taken up mainly by the Na^+^-dependent amino acid cotransport system B^0^ across the apical membrane of the epithelial cells of the intestine [27]. BCAAs in the form of di- and tripeptide forms are also incorporated by the peptide transporter PEPT1 encoded by SLC15A1. The superscript in the name of the transporter system class indicates the charge of the amino acid, with zero being used for transporters acting on neutral amino acids. The SLC6A19 gene encodes the B^0^AT1 protein, which was first detected in the brush-border membrane vesicles of the intestine [28], but this protein is also present in the proximal kidney tubule in order to reabsorb compounds from primary urine.

Mutation of SLC6A19 is reported to be the cause of the Hartnup disorder, which is an autosomal recessive inherited nutritional disorder that is manifested by a decrease in the absorption of neutral amino acids from the gut and kidney [29,30]. These mutations are credited with a clinical spectrum that ranges widely from neutral aminoaciduria, a photosensitive pellagra-like skin rash, to cerebellar ataxia, but there have been no reports specifically associating these disorders with BCAA metabolism. Nevertheless, glucose tolerance and insulin sensitivity are improved in SLC6A19 knockout mice that show malabsorption of BCAAs from nutrients [31]. This is in agreement with declines in insulin secretion via a decrease in dietary BCAAs in humans [32]. Inhibition of B^0^AT1 also suppresses the absorption of amino acids other than BCAAs, which could disrupt nutritional balance.

A Leu-preferring transporter, referred to as system L (L1), is composed of protein subunits LAT1 encoded by SLC7A5 and 4F2hc encoded by SLC3A2. System L1 transports glutamine and some amino acids in opposite directions across the plasma membrane [33]. While B^0^AT1 is present in a limited number of organs and cells, LAT1 is expressed in most cells, where it plays a pivotal role. LAT1 is a bidirectional transporter and is present in the basolateral membrane, where it secretes BCAA into the bloodstream. LAT1 is also a major transporter for the uptake of BCAAs from extracellular fluid in many nonepithelial cells such as muscle, liver, adipose tissue, and pancreatic β-cells. While LAT1 is the main transporting subunit, 4F2hc is an ancillary subunit. The SLC7 gene subfamily has 13 members, and six proteins encoded by SLC7 gene members form a heterodimer with 4F2hc via a disulfide bridge. Hepatocytes uptake BCAAs from the portal vein via LAT1, but due to low transaminase activity, most BCAAs are delivered to other organs, largely muscular tissues, without being catabolized [3,5]. LLGL2, a scaffolding protein that regulates the establishment of apical-basal polarity in epithelial cells and controls the cell surface levels of LAT1, promotes Leu uptake and the proliferation of cells—notably, breast cancer cells expressing estrogen receptors [34]. Because BCAA metabolism is robustly associated with cancer malignancy, the expression of LAT1 largely affects the efficacy of cancer treatments [35,36].

BCAAs taken up by cells are transported into mitochondria, where they experience further metabolism. Skeletal muscle, liver, and white adipose tissues are dominant organs that metabolize BCAAs or their metabolites. A recent study revealed that, in fact, brown adipose tissue (BAT) exhibits a high level of BCAA-metabolizing activity [37]. The active BCAA metabolism is facilitated by their transport into mitochondria via mitochondrial BCAA carriers (MBC) encoded by SLC25A44 [38,39]. Although lipid catabolism in BAT is considered to contribute to the generation of heat via the mitochondrial uncoupling of proteins (UCPs), BCAA catabolism increases the production of nonessential amino acids such as glutamate (Glu), alanine (Ala), and aspartate (Asp) as well as glutathione, and thus acts as a nitrogen and carbon source for their production during fasting [40].

### 2.2. Roles of Amino Acids in Maintaining Glucose Homeostasis Through Gluconeogenesis in the Liver

**Figure 2:**
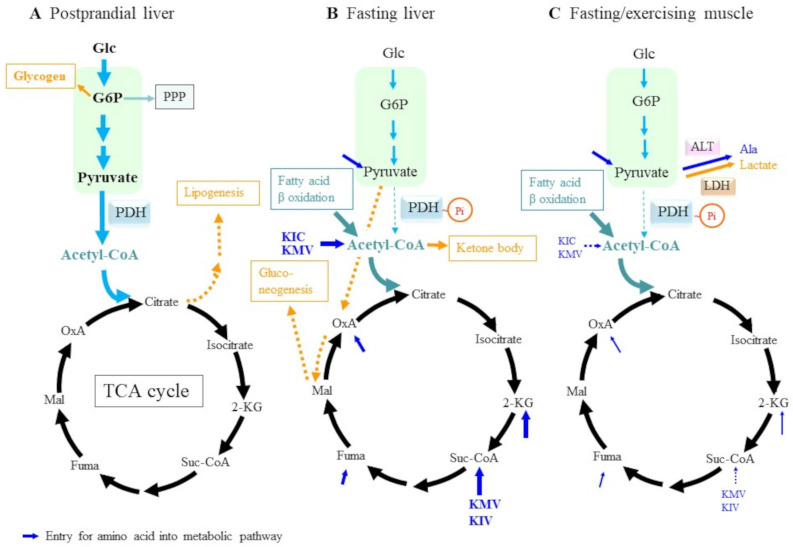
Remodeling of glucose homeostasis under nutritional conditions and in different organs. (**A**) Blood glucose becomes high (~20 mM glucose) under postprandial conditions. Glucose is effectively metabolized through glycolysis and the subsequent TCA cycle, while excessive glucose is recruited to several branched pathways at the point of glucose-6-phosphate (G6P). Glucose-6-phosphate is either isomerized to glucose-1-phosphate and recruited to synthesize glycogen synthesis or is metabolized through the glucuronate pathway. Glucose-6-phosphate is also metabolized through the pentose phosphate pathway (PPP), which results in the production of important intermediary compounds such as NADPH and ribose-5-phosphate. (**B**) Shortly after glucose is consumed, degraded hepatic glycogen becomes blood sugar. However, under long periods of fasting, the glucose supply from nutrients decreases to basal levels (~5 mM). Therefore, fatty acids and amino acids are recruited as major energy sources. The β-oxidation of fatty acids produces a huge amount of acetyl-CoA, which experiences either further degradation through the TCA cycle or is recruited for the synthesis of ketone bodies. While glucogenic amino acids experience gluconeogenesis to produce glucose, acetyl-CoA derived from fatty acids and ketogenic amino acids are both converted to ketone bodies. Due to a lack of BCAT, the liver cannot directly metabolize BCAAs, but BCKAs, which are released from the muscle, can be used for energy production, gluconeogenesis, and/or ketone body synthesis. (**C**) When ATP is consumed during exercise, creatine phosphate is first utilized to regenerate ATP. Like many other cells, glycolysis and the subsequent TCA cycle actively metabolize glucose as well as other intermediary organic compounds. Although muscular cells also store glycogen, the more intensely an animal exercises, the faster its muscle glycogen stores will be depleted. Then, in addition to blood glucose, amino acids, preferentially BCAAs derived from protein degradation and fatty acids are largely utilized for the catabolic reactions. During extreme bouts of exercise such as sprinting, oxygen supply to the mitochondrial respiratory chain may not proceed sufficiently. Thus, muscle cells enter a state similar to hypoxia, and pyruvate is no longer consumed effectively in the mitochondria. As a result, pyruvate derived from glycolysis largely accumulates and experiences an alternate metabolic conversion. A portion of pyruvate is converted to Ala by accepting amino groups from Glu via the catalytic action of alanine aminotransferase (ALT). Another portion undergoes catalysis by lactate dehydrogenase (LDH), which reduces pyruvate to lactate in an NADH-dependent manner. Consequently, both Ala and lactate are largely released from exercising muscles into the bloodstream. 2-KG, 2-ketoglutarate; Green shade, glycolysis pathway; Blue arrays, directions of amino acid metabolism; Glc, glucose; PDH, pyruvate dehydrogenase; Suc-CoA, succinyl-CoA; Fuma, fumarate; Mal, malate; OxA, oxaloacetate; Pi, phosphate group; KIC, 2-ketoisocaproate; KMV, 2-keto-3-methylvalerate; KIV, 2-ketoisovalerate.

Carbohydrates and amino acids, along with essential fatty acids, must be supplied in a balanced manner and properly metabolized to build animal bodies and support physiological functions. The liver is the central organ that directly regulates the nutritional state of the entire body by supplying blood glucose, lipids, and amino acids. Glucose is the primary carbon source for energy production through glycolysis and the coupling of tricarboxylic acid (TCA) with the electron-transport chain (ETC) in mitochondria along with a variety of anabolic reactions in daily life (Figure 2A). Glucose metabolism through the pentose phosphate pathway (PPP) produces NADPH, which provides a reducing equivalent to many anabolic reactions such as lipid and nucleotide syntheses and is also involved in the reductive recycling of oxidized thioredoxin, glutathione, and carbonyl compounds [41]. The carbon backbones of amino acids are recruited to generate glucose through gluconeogenesis, while they partially result in the production of ketone bodies. By contrast, amino acids also are a primary source of nitrogen for the synthesis of nucleotides, polyamines, and bioactive amines. Before considering the roles of BCAAs in carbon metabolism, herein, we will briefly discuss changes in the central catabolic pathways involved in energy production and their regulation under different nutritional conditions. For details on the central metabolism of glucose related to this issue and on the remodeling of its metabolic pathways in response to environmental conditions such as stress, please refer to recent reviews [42,43].

After a meal, blood glucose content elevates to ~20 mM in healthy individuals. Excess glucose can be used to synthesize glycogen in the liver and muscles via a branched pathway from glucose 6-phosphate, which is the first intermediate in glycolysis. Pyruvate dehydrogenase (PDH) is the key enzyme that continues the further oxidation of pyruvate by converting it to acetyl coenzyme A (CoA) and generating NADH and carbon dioxide [44]. Lipogenesis is also stimulated mainly in the liver, and the resultant fatty acids can be used to synthesize cellular components such as phospholipids, which are the main constituents of the biological membranes of organelles. Excessive portions of phospholipids are stored in the form of triacylglycerol in adipose tissue by transportation via very-low-density lipoprotein (VLDL). Acetyl CoA is converted to citrate by the citrate synthase in the TCA cycle. Excessive citrate is exported out of the mitochondria and subjected to a resynthesis of acetyl-CoA, which then becomes a substrate for fatty acid and cholesterol synthesis. Under energy-rich conditions, mTOR senses nutritional status, notably amino acids, and promotes multiple anabolic reactions, which include protein synthesis [45,46,47]. In this context, amino acids are employed mostly to build proteins but could partially be recruited to synthesize other nitrogen-containing compounds, such as nucleic acids. From an energetic aspect, Leu is a well-known activator of mTOR that responds to diverse environmental changes and controls many processes required for situations ranging from energy consumption to protein synthesis [48,49,50]. Sestrin 2 [51] and SARB1 [52] in the mTORC1 pathway sense Leu and transmit the signaling. In line with this, Leu administration to rats promotes mTOR complex 1 (mTORC1) activity and reduces food intake [53]. How Leu activates mTOR is an important issue in proteostasis and sports medicine and, hence, has been extensively studied, so readers should refer to review articles for further elucidation [5,46,54].

Dietary glucose is consumed after several hours, and then the basal level of blood glucose is maintained primarily by the degradation of hepatic glycogen. Subsequently, gluconeogenesis occurs in the liver mainly via the utilization of amino acids and lactate from muscle tissue (Figure 2B). Upon stimulation with glucagon, which is elevated under hypoglycemia, lipids stored in adipose tissue in the form of triacylglycerol are hydrolyzed by hormone-responsive lipase into fatty acids and glycerol, which are also utilized for energy production. Fatty acids that are transported to organs via blood in the albumin-bound form undergo β-oxidation and abundantly release acetyl-CoA within the mitochondria of aerobic cells, such as muscle and liver.

Under low glucose concentrations, pyruvate dehydrogenase kinases (PDK) phosphorylate PDH, which results in their inactivation [44]. Thus, pyruvate derived from lactate and some amino acids are efficiently recruited to pyruvate carboxylase, which results in oxaloacetic acid (OxA) and eventually glucose through gluconeogenesis. Many intermediary compounds derived from amino acid catabolism are also metabolized through the TCA cycle. Since the β-oxidation of fatty acids largely produces acetyl-CoA, excessive portions that are beyond the capacity of the TCA cycle are converted to ketone bodies in the liver mitochondria. Although neuronal cells cannot directly metabolize fatty acids, they can utilize ketone bodies to compensate for glucose insufficiency. Thus, under fasting conditions, glucose, amino acids, fatty acids, and ketone bodies are utilized as energy sources to different extents depending on the metabolic capacity of the cells.

Muscles synthesize actin and myosin from amino acids, primarily BCAAs, which constitute the mechanistic structure required for contraction. Creatine phosphate is characteristically synthesized during a resting period and is preserved at levels that are twice as high as that of ATP. Under conditions of fasting and sustained exercise, creatine phosphate is first consumed to recycle ADP to ATP by creatine kinase. Muscular cells, notably fast-twitch fiber, also called white muscle because of their low mitochondrial content, mostly derive energy from the glycolysis of glucose-1-phosphate from glycogen and release a large amount of lactate. As glycogen decreases, proteolysis is activated, and amino acids are released to provide an energy source [4,5]. Some amino acids become an energy source for muscle cells, whereas the remaining amino acids or their metabolic products are transported to the liver and used either for gluconeogenesis or ketogenesis.

A decline in blood glucose increases glucagon secretion from pancreatic α-cells, which stimulates hormone-sensitive lipase in adipose tissue. As a result, fatty acids and glycerol are released into the bloodstream to become a source of supplementary energy. Fatty acids taken up by muscles serve as another energy source for muscular tissues. Slow-twitch muscle fiber, also known as red muscle fiber because of its high content of iron-rich mitochondria, largely depends on energy from the β-oxidation of fatty acids, although a certain extent of glucose is required for efficient fatty acid catabolism through the TCA cycle [55]. Different from the liver, which synthesizes ketone bodies from excess acetyl-CoA derived through fatty acid β-oxidation, muscle uses all acetyl-CoA for energy production (Figure 2C). High concentrations of acetyl-CoA and NADH stimulate PDH kinase, which then inactivates PDH via phosphorylation and results in the preservation of pyruvate [44]. Since muscle does not perform gluconeogenesis, excess pyruvate can either be reduced to lactate by lactate dehydrogenase (LDH) or converted to Ala by alanine aminotransferase (ALT). The resultant Ala and lactate are then recruited to the liver via the bloodstream and converted to glucose through gluconeogenesis. This metabolic coordination between muscle, liver, and adipose tissue is advantageous from an energetic aspect because it can suppress the consumption of glucose and amino acids in muscle and preserve these substrates for other organs that require glucose as an exclusive energy source—most notably the brain.

### 2.3. Primary Roles of the AMPK-FOXO Axis in Protein Degradation in Muscle and Gluconeogenesis in the Liver During Fasting and Sustained Exercise

Two major systems are involved in protein degradation, autophagy [6,56] and UPS [57], and these are mechanisms well recognized for protein catabolism in muscle as well as in other organs. Ubiquitin is a highly conserved small protein that covalently binds the ε-amino group of the lysine side chain in target proteins via isopeptide bonding. At this point, polyubiquitinated proteins undergo ATP-dependent degradation via the proteasome. Enzymatic activities required for ubiquitination involve the ubiquitin-activating enzyme E1, ubiquitin-conjugating enzyme E2, and ubiquitin ligase E3 [8,9]. UPS preferentially degrades misfolded, defective, or redundant proteins but also intact proteins when required. Deubiquitinating enzymes remove ubiquitin moieties from misubiquitinated proteins and counteract excessive protein degradation. Thus, the balance between ubiquitination and deubiquitination is robustly involved in determining proteostasis. On the contrary, autophagy is a process by which cellular components, including large protein aggregates and organelles, are degraded by coupling with lysosomal function [58,59].

mTOR and adenosine monophosphate-activated protein kinase (AMPK) are two primary sensors for nutrients and energy in cells [60,61]. After a meal, mTOR is activated in the presence of abundant amino acids and insulin, which promotes protein synthesis while suppressing protein degradation via the inhibition of autophagy [45].

**Figure 3:**
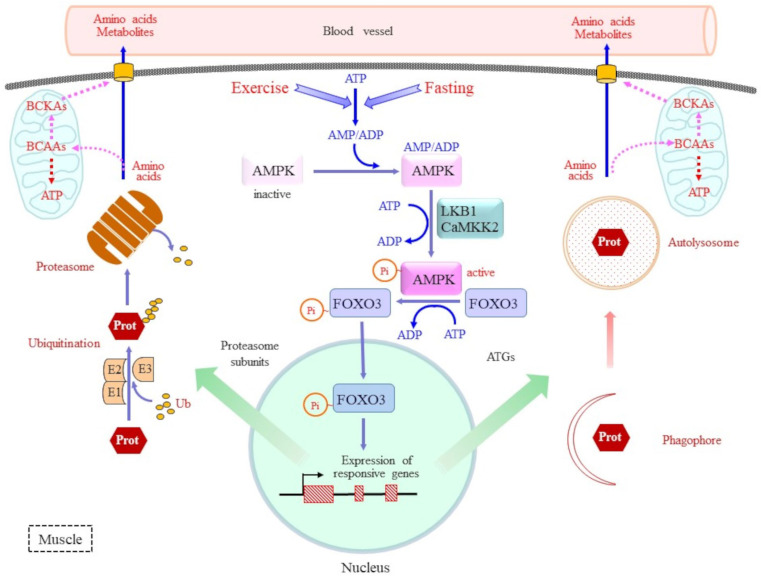
Regulatory mechanisms of protein degradation in fasting and exercising muscles. While the glucose supply is restricted under fasting conditions, ATP consumption is accelerated during exercise. Both fasting and exercise increase the ratios of AMP and ADP to ATP. The binding of AMP and/or ADP to inactive AMPK causes a conformational change that allows its phosphorylation by LKB1 and CaMKK2. Activated AMPK catalyzes the phosphorylation of the transcriptional regulatory factor FOXO3 (Ser413 and Ser588), which results in its translocation to the nucleus. As a result, the expression of genes involved in autophagy and the UPS is induced, and protein degradation progresses. A portion of the released amino acids, preferentially BCAAS, is further metabolized in muscular cells, and the remaining amino acids are released into the bloodstream. Genes responsible for protein degradation through the activation of the UPS and autophagy are induced mainly in a FOXO3-dependent manner. The resultant amino acids, including BCAAs, are partially catabolized in muscle and also largely released into the bloodstream. BCKAs, AMPK, LKB1, CaMKK2, FOXO3; Ub, ubiquitin; Pro, protein; E1, ubiquitin-activating enzyme; E2, ubiquitin-conjugating enzyme; E3, ubiquitin ligase.

Energy starvation is a common stimulus that promotes the degradation of proteins, as typically observed in muscle (Figure 3), and that of triacylglycerols in adipose tissues. AMPK is responsible for the promotion of protein degradation in muscle, which includes the induction of genes responsible for the proteolytic machinery to recruit proteinaceous amino acids for this catabolic reaction [62,63,64]. Concerning the regulation of AMPK activity, the elevation of the AMP/ATP and ADP/ATP ratios activates AMPK [65]. AMPK is composed of three subunits, with the α-subunit acting as a catalytic component and others as its regulators. Elevated levels of AMP and ADP stimulate their binding to the γ-subunit. This induces a conformational change in the α-subunit, which promotes its activation through the phosphorylation of threonine 172 (Thr172) by upstream kinases, namely liver kinase B1 (LKB1) and calcium/calmodulin-dependent protein kinase 2 (CaMKK2) [64]. Activated AMPK phosphorylates proteins to promote catabolic reactions, such as glucose uptake, ketogenesis in the liver, and fatty acid oxidation in skeletal muscle [65].

Forkhead box class O transcription factors (FOXOs) are primary transcription factors that act in muscle proteostasis by regulating the transcription of responsible genes. FOXO3 stimulates muscular protein degradation by promoting UPS and autophagy while suppressing protein synthesis [66]. Under hyperglycemia with sufficient energy sources, insulin and growth factors stimulate protein kinase B (AKT), which inhibits the action of FOXO3 via phosphorylation at Thr32 and Ser253 and recruits it for degradation by UPS [8]. However, upon energy consumption, other amino acid residues in FOXO are phosphorylated by AMPK, which leads to its stabilization. For instance, the phosphorylation of FOXO3 at Ser413 and Ser588 by AMPK promotes the translocation of active FOXO3 to the nucleus instead of its degradation [62,65]. In turn, FOXO3 upregulates the transcription of genes acting in protein degradation, which includes several ATG genes used for autophagy machinery and proteasome subunits. FOXO1 is involved in glucose production by linking retinoid metabolism to hepatic gluconeogenesis [67]. Peroxisome proliferative activated receptor-γ coactivator 1 (PGC-1α) is a transcriptional coactivator that binds and coactivates FOXO1 and enhances the expression of gluconeogenic genes in hepatic cells [68]. PGC-1α also appears to mediate the upregulation of BCAA-metabolizing genes by estrogen-related receptor-α in primary human myotubes [69]. By contrast, insulin suppresses gluconeogenesis via the phosphorylation of FOXO1 by AKT.

## 3. Enzymes Involved in BCAAs Catabolism and Their Regulation

**Figure 4:**
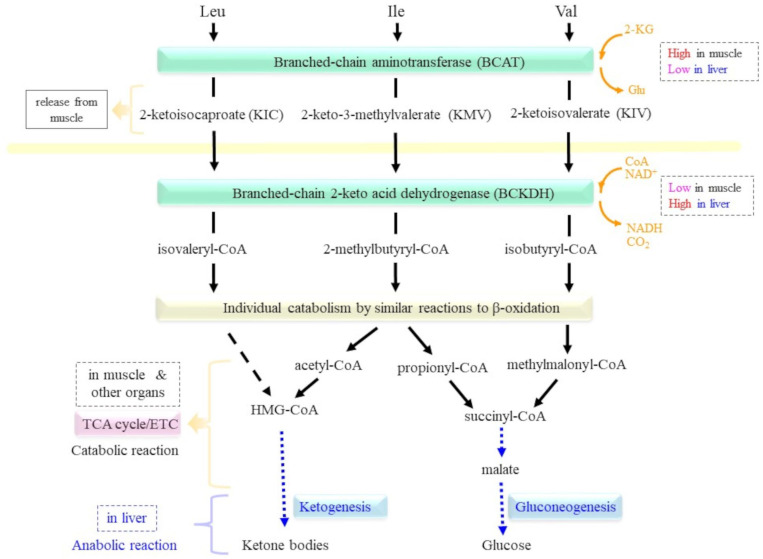
Differential metabolism of BCAAs between muscular cells and hepatocytes. BCAT commonly acts on Leu, Ile, and Val to transfer their amino groups to 2-KG in order to generate the corresponding BCKAs along with Glu. This first process occurs dominantly in muscle cells. Some BCKAs are further catabolized in muscle cells, but a large portion is secreted into the bloodstream and taken up into other cells—primarily hepatocytes. Inside cells, BCKDH catalyzes the dehydrogenation of BCKAs, which results in their Co-A conjugates. Each acyl-CoA compound undergoes further bouts of catabolism, which are reactions similar to the β-oxidation of fatty acids. Up to this point, the same process proceeds in both muscle cells and hepatocytes. In muscle and most other cells, the resultant intermediate compounds are degraded through the TCA cycle and used for energy production. However, the resultant acyl-CoA is utilized for ketogenesis and gluconeogenesis depending on the chemical forms in the hepatocytes, and the resultant ketone bodies and glucose are secreted and serve as energy sources for other organs. Therefore, the area above the thick yellow line primarily contains decomposition reactions taking place in muscle cells, while the area under the line contains both catabolic reactions in muscle and other organs, as well as anabolic reactions that proceed in hepatocytes. TCA, tricarboxylic acid; ETC, electron transport chain; CoA, coenzyme A; HMG, hydroxymethylglutaryl.

Amino acid catabolism is generally initiated by transferring the amino group to 2-ketoglutarate (2-KG) by means of aminotransferase unique to the donor amino acid, which results in a corresponding 2-keto acid from the donor amino acid and Glu from 2-KG. However, BCAA catabolism represents a slightly different picture that is not seen in other amino acids (Figure 4). While the first process catalyzed by BCAT and the second process catalyzed by BCKDH are common to all three BCAAs, the expression of these enzymes is not coordinated in the main BCAA-metabolizing organs, which are the muscle and liver [3,4]. The characteristic catalytic pathways and gene expression appear to allow BCAA metabolism to play unique roles in individual organs in response to nutritional and physical activity, which contributes to the diverged functions of BCAAs.

### 3.1. Functional Significance of the BCAT-Catalyzed Transamination of BCAAs

The first step of BCAA catabolism is the transamination of their amino groups to 2-KG catalyzed by BCAT, which requires pyridoxal-5-phosphate (PLP), a vitamin B6 derivative, for catalytic activity. As a result, BCAAs are converted to corresponding BCKAs—i.e., Leu to 2-ketoisocaproate (KIC), Ile to 2-keto-3-methylvalerate (KMV), and Val to 2-ketoisovalerate (KIV) [24,70,71]. The enzyme reaction of BCAT is reversible, so the production of amino acids from BCKAs occurs depending on the metabolite balance. Since mammals cannot substantially synthesize BCAAs due to a lack of the enzymes responsible for producing their carbon backbones, BCAT generally catabolizes BCAAs to BCKAs.

Two genes, BCAT1 and BCAT2, encode cytosolic and mitochondrial isozymes, respectively, in humans [72]. BCAT1 is expressed in a limited number of organs or tissues, such as neuronal tissues (brain, spine, and retina), reproductive organs (ovary, testes, and placenta), and pancreas, and appears to act in cell type- or tissue-specific manners [73]. For instance, BCAT1 is the predominant isoform in human macrophages [74]. A Leu analog, ERG240, blocks BCAT1 activity and reduces oxygen consumption and glycolysis along with a decrease in immune regulatory gene 1 (IRG1) expression. Since itaconate is produced from iso-aconitate in the TCA cycle by means of IRG1 and acts as a pivotal inflammatory mediator, BCAT1 appears to promote the inflammatory responses of activated macrophages.

By contrast, BCAT2 is expressed in most tissues and is found in particularly high levels in skeletal muscle, the colon, and kidneys, while the levels are quite low in the liver [37]. Muscle is the organ that shows the highest activity of BCAT, but the activity of BCKDH is low. Therefore, BCKAs that originate from muscular BCAAs are mostly released into the bloodstream, and only a portion of them is used by the muscles. BCKAs are also excreted by the monocarboxylate transporter 1 (MCT1) in cells such as glioblastoma, and they appear to modulate macrophage phenotypes [75]. BCKAs taken up via the transporter contribute to maintaining energy status and additional functions in competent cells. SMCT1 encoded by SLC5A8 and SMCT2 encoded by SLC5A12 are sodium-coupled monocarboxylate cotransporters for BCKAs as well as for L-lactate, short chain fatty acids, and ketone bodies [76,77]. SMCT2 expression is limited to kidneys, but SMCT1 is expressed systemically, which includes the brain.

Since the BCAT reaction is coupled to the conversion of 2-KG to Glu, Glu metabolism is also an important issue for controlling BCAT activity. Glutamate dehydrogenase (GDH) converts Glu to 2-KG, which simultaneously produces ammonia and NADH [78,79]. Thus, GDH, as well as NAD^+^, may indirectly affect BCAT activity. GDH is a housekeeping enzyme and is constitutively present in the cells. GDH activity is regulated by several allosteric regulators—ADP and Leu are positive regulators, and GTP and NADH are negative regulators [78,79]. Leu and ADP, which are elevated under periods of energy insufficiency in cells, bind and coordinately activate GDH. This enables the prompt provision of 2-KG to the aminotransferase reaction of BCAT, which stimulates the catabolism of BCAAs. The significance of the co-stimulation of BCAT and GDH reactions can be seen in hyperinsulinism hyperammonemia syndrome, which is caused by activating mutations in GLUD1 that encode GDH isozyme [80]. In pancreatic β-cells, the activation of GDH stimulates the conversion of Glu to 2-KG together with the production of ammonia. Then, the increased levels of 2-KG, together with BCKAs, promote ATP production via the TCA cycle coupled with the ETC, which leads to insulin release. For the same mechanism, the allosteric activation of GDH by Leu could also be responsible for insulin resistance and for the condition of type II diabetes that is associated with elevated BCAA [81,82].

Aberrant expression of BCAT is also associated with multiple pathological conditions such as neurological diseases and cancer [83]. However, the enzymatic regulation of BCAT itself and coordination with other downstream enzymes is largely unknown. Under some pathological conditions, BCAT may reverse the amino-transferring reaction. Overexpression of BCAT normalizes BCKA accumulation via reversible conversion from BCKAs to BCAAs in non-hepatic cells, which protects mitochondrial pyruvate carriers and alleviates glucose intolerance [84]. The BCAT-mediated reversible reaction appears to play important roles, notably in some cancer cells [85,86] as well as in hematopoietic stem cells and cancer stem cells [83].

A valuable resource for geneticists is the Amish population, because of a unique genetic makeup that is the result of a closed population. A genome-wide association scan (GWAS) of members of the Amish population revealed candidate chromosome loci associated with type 2 diabetes, which includes the BCAT1 gene, although none of these genes reached genome-wide significance [87]. In BCAT2-knockout mice, blood BCAA concentrations are increased more than 10 times, while adiposity and body weight are decreased, which is likely due to increased energy expenditure [88]. A recent study showed that BCAT2 physically interacts with BCKDH in mitochondria, and, in the absence of BCAT2, BCKDH activity is also abolished, which results in the accumulation of BCKAs as well as BCAAs [89]. BCAT1, in turn, reverses the morbidity and mortality caused by BCAT2 deficiency through BCKA re-amination. These observations suggest that BCKAs are directly involved in the pathogenesis of BCAT2 deficiency.

### 3.2. The BCKDH Reaction as a Key Regulator of BCAA Metabolism

Under fasting conditions and exercise, BCAAs and BCKAs that are largely released from skeletal muscle into the bloodstream are absorbed by the liver and other organs and experience further metabolism there. Although the liver rarely catabolizes BCAAs due to the low expression of BCAT, it is the primary organ that utilizes BCKAs, mainly for the purposes of gluconeogenesis and ketogenesis [3,4]. Oxidative decarboxylation of BCKAs is performed by the BCKDH complex, which releases NADH and carbon dioxide and conjugates the remaining carbon backbones to coenzyme A (CoA) to generate the corresponding acyl-CoA conjugates. The resultant acyl-CoA experiences further catabolism on an individual basis, which is similar to reactions to the β-oxidation of fatty acids [90]. BCKAs are dominantly utilized to produce energy in most organs, whereas those recruited to the liver largely experience anabolic reactions. Intermediary compounds of BCKAs metabolism, such as acetyl-CoA and succinyl-CoA, are synthesized into ketone bodies and glucose, respectively [3,4]. The resultant ketone bodies and glucose are released into the bloodstream and are taken up by organs such as the brain and skeletal muscle, where they are catabolized to produce ATP [7,91].

**Figure 5:**
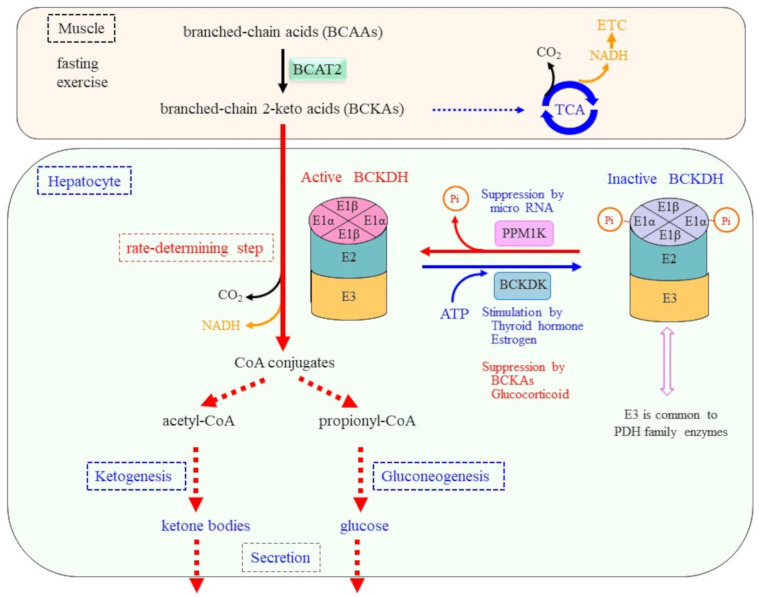
Regulation of the metabolic flow of BCKAs in the process catalyzed by BCKDH in hepatocytes. During fasting and exercise, BCAAs are largely converted to BCKAs by BCAT, which is abundantly present within muscle. While BCKAs, in part, are further degraded inside muscular cells due to low BCKDH activity, they are largely secreted. The liver is the main organ that uptakes BCKAs and further metabolizes them. BCKDH composed of E1, E2, and E3 is, in fact, present in multimers and catalyzes the rate-determining reaction process from BCKAs to acyl-CoA conjugates. It can be regulated via several mechanisms. While the phosphorylation of BCKDH E1α (BCKDHA) by BCKDK renders BCKDH inactive, the dephosphorylation by PPM1K activates it. Humoral factors such as thyroid hormones and estrogen are stimulatory, but glucocorticoid and BCKA substrates suppress the BCKDK activity. By contrast, several micro RNAs are known to suppress PPM1K protein production. BCKDH promotes the conversion of BCKAs to CoA conjugates, which are further metabolized by corresponding enzymes to intermediate compounds. While acetyl-CoA, derived from Leu and partially from Ile, undergoes ketogenesis, propionyl-CoA, derived from Val and partially from Ile, undergoes gluconeogenesis, resulting in ketone bodies and glucose, respectively. Once they are produced, both ketone bodies and glucose enter the bloodstream and are taken up and utilized as energy sources by other organs, such as the brain and muscle. E1α refers to the thiamine-dependent decarboxylase α-subunit and also is referred to herein as BCKDHA; E1β refers to thiamine-dependent decarboxylase β-subunits that herein are referred to as BCKDHB; CoA, coenzyme A; BCKAs, BCKDH, BCKDK, PPM1K, E2, dihydrolipoyl transacylase; E3, dihydrolipoyl dehydrogenase; PDH, pyruvate dehydrogse; Pi, phosphate group.

The reaction catalyzed by the BCKDH complex is the rate-determining step for BCAA catabolism and is regulated by several humoral and metabolic factors. The BCKDH complex belongs to a family of enzymes that include PDH and 2-oxoglutarate dehydrogenase (OGDH) in the TCA cycle and exhibit similar subunit composition and reaction processes [92]. In fact, the structure and reaction mechanism of the BCKDH complex have been elucidated with reference to the PDH complex, which is the most characterized family member. The BCKDH complex is located in the inner mitochondrial membrane and is composed of three types of catalytic components, which are referred to as E1, E2, and E3 [4,70]. It is noteworthy that the simple designations E1, E2, and E3 are often used to refer to individual enzymes in enzyme complexes or systems, as already mentioned for UPS above, but these abbreviations are only idiomatic and are applicable to individual enzyme systems for convenience. In the case of the BCKDH complex, branched chain α-keto acid decarboxylase (E1) is a tetrameric enzyme consisting of α2β2-subunits that catalyze the oxidative decarboxylation of BCKAs (Figure 5). The α- and β-subunits are encoded by the BCKDHA and BCKDHB genes, respectively. Dihydrolipoamide branched chain transacylase (E2), which is encoded by the DBT gene and consists of a single subunit with prosthetic lipoic acid that, in turn, transfers the acyl group to CoA. Dihydrolipoamide dehydrogenase (E3) encoded by the DLD gene is an FAD-dependent enzyme that finally transfers the released electrons to NAD^+^ and produces NADH through FAD. E3 is, in fact, a communal component of the PDH family of enzymes [93,94]. The bovine PDH complex is composed of 30 units of E1α2β2-tetramers, 60 units of E2-subunits, and 12 units of E3 dimers, which are arranged on the E2 core, and hence BCKDH is also thought to form similar large multimers [92].

Paradoxically, the liver contains only small amounts of BCAT yet is the richest source of BCKDH among all organs. This is in sharp contrast to skeletal muscle, which is rich in BCAT but has only trace amounts of BCKDH. Considering BCAAs as both primary muscle-building amino acids and a pivotal energy source under fasting/exercise in the body, the abundant expressions of BCAT and BCKDH in muscle and liver, respectively, appear to have evolved to coordinate the metabolism of BCAAs in response to high and low nutrition situations. Unlike other organs that primarily use BCKA as an energy source through the TCA cycle, in the liver, BCKAs are primarily resynthesized into glucose and ketone bodies through corresponding anabolic reactions [95]. Aberrant BCAA metabolism in brown adipose tissues could be associated with diet-induced obesity and glucose intolerance [96]. In the presence of a proteasomal inhibitor, the ablation of DBT activates autophagy through an AMPK-dependent mechanism, which leads to changes in the metabolic state of neurons and is responsible for neurodegenerative diseases [97]. Thus, DBT, as an essential component of BCKDH, could maintain protein homeostasis via the control of BCAA metabolism in neurodegenerative diseases.

In response to a variety of physiological stimuli that include nutritional, humoral, and neuronal factors, PDH plays crucial roles in regulating glucose metabolism by linking the glycolytic pathway in the cytosol to the TCA cycle in the mitochondria. Among these regulatory mechanisms, the primary regulation of PDH activity is carried out by phosphorylation/dephosphorylation. PDH kinase inactivates PDH via phosphorylation, and PDH phosphatase inversely activates PDH by dephosphorylation [98]. In a similar manner, the BCKDH activity is regulated mainly by the phosphorylation state, which depends on nutritional conditions [4]. The phosphorylation state of the BCKDH E1-subunit is determined by the coordinated action of branched chain keto acid dehydrogenase kinase (BCKDK, also designated as BDK) and a mitochondrial matrix-targeted, Mg^2+^/Mn^2+^-dependent protein phosphatase, PP2Cm, that is encoded by PPM1K. BCKDK phosphorylates at Ser337 on BCKDHA (also designated as the E1α-subunit), which inhibits the flux of BCKAs. Due to the structural and functional similarities between PDH and BCKDH, BCKDK reportedly complements the deficiency of pyruvate dehydrogenase kinases (PDK1-4) caused by gene deletion in mice [99]. Whether PDK1-4 can conversely compensate for the deficiency of BCKDK remains to be confirmed, but this is thought to be likely. Since substrate specificity of protein kinases/phosphatases may exhibit redundancy, care must be taken when employing genetically modified animals and cells to associate the functions of the targeted enzymes and phenotypes.

### 3.3. BCKDK Negatively Regulates BCKDH via the Phosphorylation of BCKDHA

The balance between BCKDK and PPM1K activities is considered to be the primary determinant of BCKDH activity. Skeletal muscle cells express the highest levels of BCKDK despite containing low levels of BCKDH, whereas the liver expresses BCKDK at very low levels [100]. That arrangement appears to rationalize the roles of BCAAs in both organs. In skeletal muscle, BCKDK phosphorylates BCKDHA and inactivates BCKDH, which potentially prevents the degradation of BCKAs derived from proteins and other sources. Then, BCKAs that are preserved due to inactivated BCKDH may be re-aminated by BCAT, which makes more BCAAs available for protein synthesis. In the case of the liver, an absence of BCKDK leads to the constitutive activation of BCKDH, which makes more BCKAs available for gluconeogenesis and ketogenesis. Moreover, BCKAs, notably KIC, allosterically suppress BCKDK, which results in recruiting more BCKAs for anabolic reactions [101].

Loss of BCKDK function by mutation is found in cases of families affected by autism, epilepsy, and intellectual disability [102]. Consistent with these findings, abnormal amino acid profiles of the brain and neurobehavioral deficits are observed in BCKDK-knockout mice. Inactive mutation in ubiquitin ligase UBE3B is the cause of a recessive neurodevelopmental disorder, Kaufman oculocerebrofacial syndrome, which is characterized by intellectual disability and a lack of speech. BCKDK is actually an in vivo substrate of UBE3B, and plasma and cortical metabolomes reveal perturbations in both nucleotide metabolism and the TCA cycle in UBE3B-deficient mice [103]. Reports have associated BCKDK mutation with diseases—most notably concerning those of the neuronal system [104,105]. A recent study was performed on the largest cohort of patients with a diagnosis of BCKDK deficiency [106]. After treatment with a high-protein diet (≥2 g/kg/day) and BCAA supplementation (100 to 250 mg/kg/day), plasma BCAA increased significantly and was accompanied by a stabilization of the motility functions of muscle and improved head circumference. The data show that if introduced early in life, a high-protein diet together with BCAA supplementation has the potential to improve neurodevelopmental outcomes.

In the absence of BCKDK, it is conceivable that BCKDH activity is not effectively suppressed; hence, the degradation of BCKAs, as well as that of BCAAs, is presumably stimulated. In fact, when BCKDK is deleted throughout the body, blood BCAA concentrations decrease by more than 50% [107]. A spontaneous mutation in BCKDK, which is associated with a decreased phosphorylation of BCKDHA, is found in Sprague-Dawley rats that share properties with knockout mice [108]. When BCKDK is specifically deleted in muscle tissue (cardiac or skeletal muscle), the level of BCAAs is reduced by approximately 30% [109]. While conditional knockout mice are healthy following the feeding of a protein-rich diet, feeding a low-protein diet causes a decrease in myofibril proteins, which can be rescued by BCAA supplementation in drinking water. These results collectively confirm that BCKDK, which is highly expressed in muscle, generally suppresses the degradation of BCAAs by inhibiting BCKDH and thereby recruiting BCAAs for building muscular proteins. Analysis of the Gene Expression Omnibus (GEO) database shows that BCKDK expression levels in skeletal muscle are consistently decreased in aging and in pathologic conditions, which include muscular diseases and interrupted muscle metabolism [100].

It is noteworthy that ATP-citrate lyase (ACL) is an alternate substrate of BCKDK and PPM1K, although phosphorylation status exerts opposing effects on its enzyme function [110]. ACL is localized in the cytoplasm and uses the energy of ATP hydrolysis to convert citrate into acetyl-CoA and oxaloacetate. This reaction provides acetyl-CoA for the synthesis of fatty acids and cholesterol. Phosphorylation of ACL by BCKDK activates lipogenesis, whereas dephosphorylation by PPM1K suppresses it. Administration of BT2, an inhibitor of BCKDK [111], or the overexpression of PPM1K, decreases the phosphorylation of ACL, which thereby suppresses enzymatic activity in the liver, but the overexpression of BCKDK increases enzymatic activity. The carbohydrate response element binding protein (ChREBP) upregulates the expression of multiple genes acting in glycolysis and lipogenesis in response to cellular carbohydrates [112]. ChREBP increases BCKDK but decreases PPM1K, which stimulates lipogenesis through the activation of ACL, regardless of BCAA catabolism [110]. A pathological condition of nonalcoholic fatty liver disease (NAFLD) may advance to nonalcoholic steatohepatitis (NASH) and hepatic cancer. Because sterol regulatory element-binding protein (SREBP)-1 also transcriptionally upregulates BCKDK, lipogenic genes induced by SREBP-1 and BCKDH suppression by BCKDK-mediated phosphorylation coordinately stimulate lipogenesis and could be involved in the pathogenesis of NAFLD [113]. Thus, the inhibition of BCKDK is potentially an effective treatment for metabolic diseases such as NAFLD/NASH and hepatic cancer, and specific inhibitors targeting BCKDK have been explored. However, inhibitors with different structures may have different outcomes by inhibiting BCKDK degradation or stabilizing it, so attention must be required before such drugs are applied in a clinical regimen [114].

### 3.4. PPM1K Positively Regulates BCKDH Activity via the Dephosphorylation of BCKDHA

PPM1K is a member of a metal-dependent protein Ser/Thr phosphatase (PPM) family and requires manganese/magnesium ions (Mn^2+^/Mg^2+^) for its activation [115]. PPM1K was first found to regulate mitochondrial membrane permeability transition pore opening [116]. Subsequently, PPM1K has been shown to dephosphorylate BCKDHA and stimulate the BCKDH activity [117]. PPM1K is highly expressed in the brain, heart, kidney, and diaphragm, as well as in the liver, but exists in lower amounts in skeletal muscle [118]. The expression of PPM1K is regulated in response to nutrient status. PPM1K expression is downregulated by several micro RNAs such as miR-22, miR-204, and miR-211 in a species-specific manner [119], although it is unclear how they are controlled by nutritional status.

PPM1K-deficient mice are lean and show changes in metabolism, such as enhanced insulin sensitivity and glucose tolerance, which are predominantly observed in the liver but neither in skeletal muscle nor in white adipose tissue [120]. Experiments employing systemic PPM1K knockout mice have demonstrated that sustained weight loss and improved glucose tolerance after vertical sleeve gastrectomy do not require a reduction in circulating BCAAs [121]. Ablation of PPM1K decreases the signaling of TALE homeodomain transcription factor MEIS1/p21 and reduces the glycolysis and quiescence of hematopoietic stem cells, which consequently has extended survival in a murine leukemia model [122]. The deficiency of PPM1K suppresses BCKDH activity and the consequent conversion of BCKA to CoA conjugates, which eventually impairs gluconeogenesis from BCKAs [84]. Heart-specific PPM1K knockout blunts the cardioprotective effects of exercise against myocardial infarction, which suggests the involvement of BCKAs in exercise-supported cardioprotection [123]. Mitochondrial pyruvate carrier is most susceptible to, and suppressed by, an elevation of BCKAs, and therefore, pyruvate metabolism in mitochondria is decreased by PPM1K deficiency, which leads to glucose intolerance. Impairment of BCAA degradation causes an accumulation of BCAAs in CD8^+^ T cells, which has led to a hyper-activation of CD8^+^ T cells and enhanced anti-tumor immunity in PPM1K-deficient mice [124]. Conditional knockout of PPM1K in the lumbar dorsal root ganglion upregulates the C-C chemokine ligand 5/C-C chemokine receptor 5 (CCL5/CCR5) and increases transient receptor potential ankyrin 1 (TRPA1) expression, which appears to be associated with mechanical allodynia—heat and cold hyperalgesia [125,126].

## 4. Roles of BCAAs and BCKAs in Pathogenesis

These are important issues in human lives and have been extensively overviewed but will not discuss pathology here. Therefore, readers should refer to relevant reviews for details [5,22,127]. Instead, herein, we discuss the current consensus on representative metabolic disorders directly associated with BCAAs and their metabolites, mainly from aspects of biochemical metabolism and gene regulation. An increase in dietary BCAAs causes obesity, type II diabetes, cardiovascular diseases, and some types of cancer; in line with these reports, their decrease tends to maintain a healthy state [13,128,129,130]. However, some debate remains as to whether BCAAs and their metabolites actually cause such diseases or whether their levels are merely increased in association with disease conditions.

### 4.1. MSUD Is Caused by Defected BCKDH Subunits

Due to the physiological significance of BCAAs, some inheritable diseases are linked to BCAA metabolism, which includes disorders affecting the metabolism of either all three BCAAs or specific to each BCAA [10,131,132,133,134]. MSUD is caused by rare inborn errors (approximately 1 in 150,000 live births) but is a significant disease because it has been linked to an aberrant metabolism of all BCAAs due to mutations in genes encoding BCKDH subunits that result in multiple abnormalities [70,133,135]. MSUD is associated with the maple syrup odor in the urine of patients, and intellectual disability is commonly observed in patients without proper treatment. Human MSUD can be classified into one of five clinical phenotypes, and ~100 mutations have been identified mostly in BCKDHA, BCKDHB, and DBT (45, 35, and 20% of MSUD patients, respectively) and to a lesser degree in DLD and other genes [10,95]. Since E3 encoded by DLD in the BCKDH complex is a communal component of the PDH family of enzymes [93,94], variants of DLD have shown a variety of aberrant phenotypes, which are not considered part of the category of typical MSUD, and as such assigned as MSUD type 3 [136]. Mutational inactivation of PPM1K reportedly causes a mild form of MSUD with elevated BCAA levels [137,138]. Moreover, mutations in BCAT2 have been detected in a few familial cases and have been associated with wide-ranging clinical phenotypes in individuals, although their properties differ from those of MSUD [88]. Concerning pathological model animals, a mutant mouse strain raised with treatment from a mutagen, N-ethyl-N-nitrosourea, has exhibited an elevation in BCAAs and clinical features that are similar to those of MSUD [89]. The symptoms are associated with a splice-site mutation in the BCAT2 gene. Concerning clinical symptoms and treatment, there are many review articles on this disease, e.g., [70]; therefore, herein we outline some of the basic aspects of how defective BCAA metabolism causes pathogenesis.

Regarding the central nervous system, large neutral amino acids, such as BCAAs and aromatic amino acids, are transferred from the blood to the brain via LAT1 through the blood-brain barrier. An abundant presence of BCAAs competitively inhibits the uptake of the aromatic amino acids phenylalanine, tyrosine, and tryptophan, which are precursors for some compounds that are essential for neuronal functions [91,139]. Accordingly, neurological abnormalities are considered a consequence of the insufficient production of such neurotransmitters. The basic treatment is to reduce the dietary intake of BCAAs, but this alone appears insufficient, and there appear to be alternative causes. So, a precise understanding of the pathways involved in their metabolism and the pathogenic mechanisms involved requires further study. Recent studies have revealed novel roles of BCKAs and their downstream metabolites in the central nervous system, so either the excessive presence of BCKAs or the decreased supply of downstream metabolites could additionally contribute to the neurological symptoms observed in MSUD [11]. For instance, elevated levels of BCKAs, notably KIC, likely reduce glutamate, glutamine, and γ-aminobutyrate (GABA) in the brain, which leads to dysfunction in the neuronal system [91,139]. KIC is also known to stimulate insulin secretion in β-cells independent of glucose [140]. In addition to a decreased production of key neurotransmitters, intracellular Met decreases in MSUD because its uptake via LAT1 competes with the abundant presence of BCAAs in the extracellular milieu. Met is a precursor for S-adenosylmethione, which is the methyl group donor for synthesizing catecholamines, polyamines, DNA, and other methylation reactions [141]. Therefore, Met deficiency could lead to a decline in their production or modification. Moreover, the sulfur in Met is used to generate cysteine via a transsulfuration reaction. Cysteine is a primary component in the synthesis of glutathione, which is an essential redox molecule that maintains the redox homeostasis of the body [142]. Accordingly, a defect in Met uptake could cause dysfunction in a variety of metabolic abilities and oxidative damage, notably in the central nervous system where the provision of glutathione from blood is prohibited due to the blood-brain barrier.

### 4.2. KLF15 Plays Contradictory Roles in Aggravating Sarcopenia While Protecting Cardiac Function

Since BCAAs are dominant constituents of muscular proteins and preferably catabolized, their aberrant metabolism has been associated with muscular diseases. Sarcopenia is often accompanied by aging or in association with diseases, notably cirrhosis, and characteristically shows a general loss of skeletal muscle mass and strength [143]. BCAAs are decreased in the sarcopenic population [144,145], and while the supplementation of BCAAs likely alleviates this symptom, other studies have reported opposite results [146,147,148,149]. Thus, there is a discrepant observation concerning the effects of BCAAs on muscular function, as also observed in insulin resistance, which appears to be largely attributed to individual differences in the genetic background and environmental factors of patients.

From the aspect of the regulatory mechanism of gene expression in association with muscle degradation, recent research has made significant progress. Crucial roles of the Kruppel-like factor-15 (KLF15), a member of the KLF family of transcription factors [150], has attracted much attention in controlling muscular homeostasis, notably under pathological conditions such as diabetes, Duchenne muscular dystrophy, and heart failure [151]. LF15 belongs to a subclass of zinc finger DNA-binding proteins that induce the expression of genes for modulating the metabolism of several nutrients, which includes BCAA. Under normal conditions, ubiquitin ligase WWP1 ubiquitinates KLF15 and thereby promotes its degradation by proteasome. However, upon hyperglycemia in diabetic model mice, a decline in the WWP1 level stabilizes KLF15 by attenuating ubiquitination [152]. The resultant aberrant activation of KLF15 is likely a causative factor for muscular atrophy by stimulating protein degradation.

BCAAs appear to negatively regulate KLF15 expression via the phosphoinositide-3-kinase (PI3K)-AKT pathway so that a decline in BCAA abundance by fasting leads to a marked induction of KLF15 expression in muscle [153]. Immobilization of mice limbs decreases Piezo1, which is a mechanosensitive cation channel and leads to a decline in cellular calcium concentration in skeletal muscle [154]. An increase in the expression of KLF15 induces interleukin-6 (IL-6) expression. While IL-6 signaling is associated with the stimulation of hypertrophic muscle growth, it also promotes atrophy and muscle wasting [155]. These findings expose that an adequate level of calcium ions must be supplied via Piezo1 for muscle maintenance and growth [156]. Thus, the molecular mechanisms of muscle protein degradation under pathological conditions are mechanistically different from those occurring during fasting and exercise, which involves the AMPK-FOXO axis, as described above. On the contrary, a recent study reports opposing data, which show an essential role of KLF15 in myoblast differentiation and muscle regeneration [157]. KLF15 induces the expression of FK506 (tacrolimus)-binding protein 51 (FKBP5), which is a positive regulator of myoblast differentiation. These collective findings show that KLF15 function must be tightly regulated because it appears to promote either muscle breakdown or regeneration, and this result likely depends on physiological situations. Concerning other KLF isoforms, KLF5 appears to be dominantly involved in the regulation of smooth muscle [150]. Nevertheless, KLF5 expression increases with aging, and sarcopenia development positively correlates with the expression of the atrophy-related ubiquitin ligase genes FBXO32 and TRIM63 [158]. Since information on KLF5 is limited, further research is needed to understand its genetic regulation concerning sarcopenia.

Cardiac hypertrophy is a pathological state that, without proper treatment, leads to heart failure. While the activation of KLF15 likely causes sarcopenia through protein degradation in skeletal muscle, accumulating evidence indicates that KLF15 suppresses cardiomyocyte hypertrophy [159,160]. This beneficial action of KLF15 on cardiac hypertrophy is the result of suppressing the accumulation of BCAAs and BCKAs [161,162]. This is accomplished by an induced expression of BCAA metabolic enzymes, such as BCAT2, BCKDH subunits, and PPM1K by KLF15 in the heart. Since BCAAs promote the hypertrophic growth of cardiomyocytes through the activation of mTOR signaling, the KLF15-mediated activation of BCAA degradation tends to prevent cardiac hypertrophy. In cardiomyocytes, high levels of glucose inhibit the cAMP response element-binding protein (CREB)-stimulated KLF15 transcription, which subsequently suppresses the expression of these BCAA metabolic enzymes and consequently causes a decline in the degradation of BCAAs [163]. Interplay concerning KLF15-Wnt dynamics defines the network control of cardiomyocyte and vascular cell homeostasis in the postnatal heart and demonstrates its potential as a cardiac-specific therapeutic target in heart failure [164]. Melatonin treatment activates the protein kinase PKGIα, which induces KLF15 through the phosphorylation of CREB [165], at which point KLF15 induces the expression of BCAT2 and BCKDH to attenuate mTOR action by degrading BCAAs. In line with this scenario, researchers conducting a study based on feeding a high-cholesterol diet to ApoE-deficient mice determined that a restriction of excessive BCAA likely alleviates atherosclerosis and subsequent coronary atherosclerotic heart disease [166]. By contrast, after myocardial infarction, the oxidation of BCAA in the body, the only exception being cardiomyocytes, tends to lower blood pressure via an increase in vascular relaxation, which results in cardioprotection, and this process is independent of nitric oxide production [167].

### 4.3. BCAA Metabolism in Association with Diabetes

Diabetes is a major metabolic disorder that is associated with complications in many organs; hence, the issues are discussed in the context of BCAA metabolism in several sessions of this paper. Elevation in the circulating levels of BCAAs and their metabolites and correlation with insulin resistance has long been known in obese patients, but despite extensive studies, the underlying mechanisms and significance of these changes have not been fully elucidated [13,14,21,90,168]. For instance, in studies using animal models fed a high-fat diet, supplementation with BCAAs exacerbated symptoms in insulin-resistant rats [169]. Consistent with these findings, BCAA restriction increases insulin sensitivity in both lean and obese animal models [128,170,171]. However, there are also reports indicating that neither increased BCAA catabolism in muscle nor lowering plasma BCAAs improves insulin sensitivity in mice [172]. Also, the pharmacological activation of BCKDH in skeletal muscle and liver does not account for the improved insulin sensitivity. These observations show that the modulation of BCAA metabolism by multiple tissues contributes to insulin sensitivity.

Insulin suppresses hepatic BCAA metabolism via the stimulation of the central nervous system [173,174]. Metformin, a glucose-lowering drug, also suppresses BCAA catabolism and increases plasma BCAA content [175]. Based on an analysis of human islets from donors, some genes have been identified as putative type 2 diabetes risk variants, and PPM1K is the gene that influences insulin secretion in INS-1 cells [176]. While obese people who might develop insulin resistance or type 2 diabetes mellitus in the future show trends of increased BCAAs in the blood, some anti-obesity effects of BCAAs have been reported—notably in rodent models. By contrast, some studies claim that increased BCAA levels are more likely to be a marker for a decline in the function of insulin rather than a cause of insulin resistance [81]. In line with this, in obese and insulin-resistant states, hepatic BCKDH is inactivated by increased BCKDK activity. Supplementation with BCAAs to a low-fat diet for extended periods (12 to 15 months) increases appetite and weight but shortens the lifespan in mice [177]. However, this appears to be caused by hyperphagia involving an interaction with tryptophan and threonine instead of intrinsic BCAA toxicity. As such, there remains no consensus on how BCAAs are related to aberrant glucose metabolism and diabetes.

### 4.4. Individual Roles of BCAA Metabolites in Physiology and Pathophysiology

While glucose produced from Leu and Ile through gluconeogenesis becomes a general source of energy for all organs, recent studies have revealed that ketone bodies produced from Ile and Val, as well as fatty acids, also play multiple roles, such as providing fuel and being messengers in various organs [178,179]. Moreover, accumulating evidence indicates that BCKAs themselves perform many more biological functions either directly or after further metabolism, which includes cellular signaling, protein modification, and the epigenetic regulation of gene expression.

Leu is the most abundant form of BCAA and has been extensively studied from the aspects of its association with exercise physiology and metabolic disorders, which has produced numerous citations in the literature concerning the metabolic actions and pathological associations of its metabolism with disorders [46,54,81]. Leu is catabolized to KIC by BCAT, which then inhibits muscle proteolysis but stimulates protein synthesis. In turn, β-hydroxy-β-methylbutyrate (HMB) produced from KIC is reported to promote muscle recovery after exercise. However, controversial results have been published, and a recent survey of the literature did not support this benefit [147,180,181]. Thus, there are inconsistencies regarding the actions of Leu in these physiological situations.

Leu and KIC suppress insulin-stimulated glucose uptake in L6 myotubes along with the activation of the pathway that involves S6 kinase 1 (S6K1), which is a downstream kinase of mTOR signaling, and insulin receptor substrate 1 (IRS1) [182,183,184]. While oral administration of Leu has induced mTORC1/IRS1 signaling in muscle and in the livers of rats, neither Leu nor KIC changes insulin sensitivity. Thus, neither Leu nor KIC appears to cause insulin tolerance in vivo, which might be caused by their metabolic removal by organs [185]. Concerning effects on the immune system, KIV exerts a pro-inflammatory effect on macrophages, but KIC and KMV lead them to a pro-tumoral state [186].

From the aspect of nutrition, low-protein diets are reportedly associated with BCAAs in promoting metabolic health in both rodents and humans. Precise experiments on mice have shown that a reduction in Ile and Val [187], or in Ile alone [188], tends to promote metabolic health in mice. In fact, Ile in a diet has been positively associated with human body mass index (BMI), and the beneficial effects of Ile restriction appear to be mediated by the FGF21-UCP1 axis [187]. Genetic errors in Ile-degrading enzyme genes have been associated with diseases such as acute episodic ketoacidosis [132]. The peptide transporter PEPT1, encoded by SLC15A1, could be associated with the production of the fibroblast growth factor 21 (FGF21), which is a humoral factor secreted from the liver and appears to modulate global insulin signaling [27].

Post-translational modification with low molecular compounds, such as methylation and acetylation, alters the function of proteins and other biological compounds. Among them, histone modification with methyl and acetyl groups dominantly regulates epigenetics. When the numbers of this type of chromatin modification increase, reactive acyl-CoA conjugates are generally used as the donor compound for the modification of the amino group [189]. Propionyl-CoA is abundantly produced during Ile metabolism, as well as minor recruitment from other pathways, and could result in the modification of lysine in chromatin, which would be associated with epigenetics [190]. While acetylation is a major modification of proteins, acetyl-CoA is dominantly produced from glucose and fatty acid catabolism, so the contribution of BCAA catabolism to acetylation might not be significant overall. Similarly, while succinyl-CoA, a substrate for the succinylation reaction, could be acquired from both Ile and Val, the TCA cycle is the most abundant provider. Among such posttranslational modifications, propionylation occurs using propionyl-CoA as a donor compound [191]. Propionylation of histone lysine, which appears to be catalyzed by acetyltransferase p300/CBP, promotes gene expression; by contrast, histone deacetylase (HDAC) 1/3 likely erases the modification [192]. Since Ile elevates histone propionylation, the resultant epigenetic change could also be associated with the metabolic events of Ile. Consistent with this notion, histone propionylation reportedly reduces pressure overload-induced hypertrophic changes in the heart [193]. Propionylation could also target proteins other than histones and alter their functions directly in a BCAA concentration-dependent manner. For instance, BCAAs enhance the propionylation of tropomodulin-3 at lysine-255 in platelets, which are involved in the integrin αIIbβ3-mediated activation of platelets as well as in thrombosis formation [194].

β-Hydroxyisobutyric acid (HIBA), a metabolic intermediate in the Val degradation pathway, along with a few other compounds, correlates with markers of adipose browning and has an inverse association with the BMI [195]. HIBA reportedly reduces adiposity, increases energy expenditure, and improves glucose and insulin homeostasis in mouse models of obesity and diabetes. By contrast, HIBA activates fatty acid uptake in vascular endothelium and skeletal muscle, which leads to an accumulation of ectopic lipids in skeletal muscle that results in insulin resistance [196]. Studies reported in the literature show that an elevation in levels of HIBA is associated with diabetes [197,198]. In addition, apart from the presence of diabetes, high levels of HIBA have been associated with a prognosis of chronic heart failure [199]. Thus, it is certain that plasma HIBA concentrations are associated with several metabolic diseases, but further investigation is needed to determine whether HIBA is beneficial or detrimental. Moreover, β-aminoisobutyric acid (BAIBA) is produced from methylmalonate semialdehyde, which is a subsequent catabolic intermediate of HIBA. BAIBA induces beige coloration of white adipocytes and β-oxidation in the liver, and its blood concentration is inversely proportional to the risk for heart disease [200]. BAIBA also attenuates insulin resistance and induces fatty acid oxidation via the AMPK-peroxisome proliferator-activated receptor-δ (PPARδ) pathway in skeletal muscle [201]. BAIBA is also known to prevent the loss of bone and muscle function in a murine hindlimb unloading model [202]. BAIBA and FGF23 are increased by exercise and coordinately maintain phosphate homeostasis [203]. As seen in these findings, previously unknown functions of not only BCAAs but also their metabolites are becoming apparent.

## 5. BCAAs in Cancer

It is well known that metabolic reprogramming occurs in cancer cells, and BCAA metabolism is also altered in various cancers and is involved in their malignant phenotypes. Several review articles have discussed the association of BCAAs with cancer [12,204,205]. BCAAs are essential for cancer cell growth via the activation of the mTOR pathway to promote protein synthesis and serving building blocks for proteins and substrates for energy production [205].

### 5.1. Increased Metabolism of BCAAs Is Frequently Observed in Gastrointestinal Cancers

Increased BCAA levels have been reported in patients with colorectal and pancreatic cancers [206,207]. On the other hand, high plasma Leu and Val levels are reportedly inversely correlated with the risk of colorectal cancer [208]. Mayers et al. [206] demonstrated that elevated plasma levels of BCAAs occur early in the development of pancreatic cancer. This phenomenon has also been observed in KRAS-driven pancreatic ductal adenocarcinoma (PDAC) cancer models but not in other cancer models in mice. It appears that the breakdown of peripheral tissues, notably muscles, may elevate BCAA levels in the blood of pancreatic cancer [206,209]. Elevated BCAA levels in the blood have also been reported in both mouse and human hepatocellular carcinoma (HCC), which has been attributed to the loss of BCAA catabolism [210]. The suppression of BCAA catabolic enzyme expression leads to an accumulation of BCAA and to the hyperactivation of the mTOR pathway in cancers. Whereas the intake of high dietary BCAA reportedly enhances tumor development and growth in mice [210], BCAA treatment improves overall survival in human HCC patients [211,212] and prevents the development of breast cancer and HCC in mice [213,214]. Thus, an increase in BCAAs in the blood and an increase due to artificial administration have different effects on cancer growth, but the reason for these differences remains unknown. Although further investigation is needed to elucidate this issue, blood BCAA levels could nonetheless serve as a predictor for prognostic indicators of cancer.

### 5.2. Elevation of BCAT Activity Is Rather Common in Cancers

Elevated BCAT1 expression is commonly observed and is associated with lower rates of survival in patients with various types of cancer [215,216,217]. Overexpression of c-Myc enhances BCAT1 mRNA levels and promotes cell invasion in nasopharyngeal carcinoma [218]. Additionally, KRAS stabilizes BCAT2 through the spleen tyrosine kinase (SYK) and E3 ligase tripartite-motif-containing protein 21 (TRIM21) [219]. These findings suggest that the regulation of BCAA-metabolizing enzymes is enhanced by oncogenic processes that play an essential role in cancer development. BCAT1 is known to support cancer cell growth in a glioblastoma model [220]. In breast cancer, elevated BCAT1 levels promote mitochondrial biogenesis in an mTOR-dependent manner, which supports cancer proliferation [221]. In HCC, BCAT1 expression is significantly elevated, and its ectopic expression enhances tumorigenic properties and confers cisplatin resistance [222,223]. BCAT converts BCAAs and 2-KG to BCKAs and glutamate, which is an initial process essential for cancer development [2]. However, the BCAT reaction is reversible, so BCAT also synthesizes BCAAs from available BCKAs. Recent reports suggest that BCAAs are synthesized by the reverse reaction of BCAT and are utilized by chronic myeloid leukemia cells [85,86]. Stromal BCAT1 induced in PDAC tumors reportedly fuels BCKA demand [224]. Furthermore, more than 60% of intracellular BCKA are known to be used for synthesizing BCAAs, which are subsequently utilized for protein synthesis in PDAC [12]. These findings indicate that BCAT1 plays an important role in tumor growth, possibly through enhancing mitochondrial metabolism and mTOR activation. Additionally, Qian et al. [225] have reported the occurrence of BCAT1^E61A^ mutation and its involvement in gastric cancer. Mutated BCAT1^E61A^ features high levels of enzymatic activity that stimulates BCAA catabolism and promotes cell growth and motility, which contributes to tumor development. In addition to enzymatic activity, BCAT1^E61A^ also interacts directly with RhoC, a subfamily of Rho GTPase, which leads to an elevation of RhoC activity. Since activated RhoC controls cytoskeletal dynamics, this finding exposes a novel function of BCAT1 concerning the direct regulation of cancer cell dynamics and association with malignant phenotypes. Although further research is needed to determine whether BCAT1/2 catabolizes or synthesizes BCAAs in cancer cells, BCAT1/2 could be a potential therapeutical target for cancer treatment.

### 5.3. BCKDK Is Elevated in Some Cancers

BCKDK has also been associated with cancer prognosis and malignancy. Elevated expression of BCKDK correlates with a poor prognosis for several cancers, such as breast cancer, colorectal cancer, and non-small lung cancer [226,227,228]. BCKDK overexpression has also been linked to advanced pathological grades in ovarian cancer [229]. Recently, BCKDK has been shown to have a novel kinase activity in addition to regulating BCAA metabolism. BCKDK reportedly promotes the tumorigenesis of colorectal cancer by upregulating the mitogen-activated protein kinase (MEK)-extracellular signal-regulated kinase (ERK) signaling pathway [230]. Nonreceptor tyrosine kinase Src appears to enhance the activity and stability of BCKDK through phosphorylation at tyrosine 246, which promotes the migration, invasion, and epithelial-mesenchymal transition of colorectal cancer cells [226]. Aminopeptidase N (also known as CD13) reportedly mediates the phosphorylation of BCKDK at serine 31 (Ser31) and activates its downstream pathway that is involved in ERK, which promotes the proliferation and metastasis of HCC [231]. Cancer cachexia is accelerated in muscle-specific BCKDK deficiency in mice after Lewis lung cancer cell transplantation [232]. Increased UPS activity and impaired protein synthesis consequently induce skeletal muscle wasting. BCKDK suppresses BCKDH activity via phosphorylation, which suggests that BCKAs are likely increased in these malignant cells. However, it remains unclear whether BCKDK promotes cancer growth primarily by suppressing BCKDH activity or through the stimulation of other kinase activity, such as ERK. There is no doubt that BCKDK plays a critical role in cancer development, but in order to develop effective cancer treatment, it is necessary to clarify which mechanism is responsible. The literature cited in the cancer session is summarized in Table 1.

## 6. Perspectives

BCAAs are important building blocks of muscle proteins and act as dominant sources of energy metabolism during starvation and exercise. BCAT and BCKDH are commonly involved in the metabolism of BCAAs, and their dysfunction is related to various metabolic disorders such as MSUD, diabetes, cardiovascular dysfunction, and cancer. Despite extensive studies on human and model animals, many unclear and contradictory issues remain. Moreover, in addition to being understood as a group, each BCAA must also be understood on an individual basis. The development of high-throughput analytical instruments could enable the comprehensive elucidation of responsible proteins and metabolites. Artificial intelligence (AI) could be effective in analyzing the huge amount of data thus obtained, which could contribute greatly to elucidating the diverse functions of BCAAs.

## Figures and Tables

**Table 1:** Representative reports on cancers related to BCAA metabolism.

Cancer	Plasma BCAA Levels	BCAT1	BCAT2	BCKDK
Acute myeloid leukemia		Increased expression [215]		
Chronic myeloid leukemia		Synthesizes BCAA from BCKA [85,86]		
Breast cancer		Supports cancer proliferation [221]		Increased expression [227]
Colorectal cancer	Increased [208]			Upragulates ERK activity [230] Phosohorylated by Src [226]
Gastric cancer		Increased expression [216] BCAT^E61A^ mutation enhances its enzyme activity [225]		Upregulates RhoC activity [225]
Glioblastoma		Supports cancer proliferation [220]		
Hepatocellular carcinoma	Increased [210]	Increased expression [217] Supports cancer proliferation [222,223]		Phosphorylated by CD13 [231]
Nasopharyngeal carcinoma		Increased expression by c-Myc [218]		
Non-small lung cancer				Increased expression [228]
Ovarian cancer				Increased expression [229]
Pancreatic cancer	Increased [206,209]	Synthesizes BCAA from BCKA [12]	Increased expression by KRAS [219]	
